# Sleep behavioral outcomes of school-based interventions for promoting sleep health in children and adolescents aged 5 to 18 years: a systematic review

**DOI:** 10.1093/sleepadvances/zpae019

**Published:** 2024-03-29

**Authors:** Cadeyrn J Gaskin, Carolina Venegas Hargous, Lena D Stephens, Gunchmaa Nyam, Victoria Brown, Natalie Lander, Serene Yoong, Bridget Morrissey, Steven Allender, Claudia Strugnell

**Affiliations:** Faculty of Health, Deakin University, Geelong, VIC 3220, Australia; Faculty of Health, Deakin University, Geelong, VIC 3220, Australia; Faculty of Health, Deakin University, Geelong, VIC 3220, Australia; Faculty of Health, Deakin University, Geelong, VIC 3220, Australia; Faculty of Health, Deakin University, Geelong, VIC 3220, Australia; Faculty of Health, Deakin University, Geelong, VIC 3220, Australia; Faculty of Health, Deakin University, Geelong, VIC 3220, Australia; Faculty of Health, Deakin University, Geelong, VIC 3220, Australia; Faculty of Health, Deakin University, Geelong, VIC 3220, Australia; Faculty of Health, Deakin University, Geelong, VIC 3220, Australia

**Keywords:** sleep behavior, children, adolescents, school, cluster-randomized trial, randomized controlled trial

## Abstract

**Study Objectives:**

Insufficient sleep is common among children and adolescents, and can contribute to poor health. School-based interventions potentially could improve sleep behavior due to their broad reach, but their effectiveness is unclear. This systematic review focused on the effects of school-based interventions on sleep behavior among children and adolescents aged 5 to 18 years.

**Methods:**

Five electronic databases were searched for randomized controlled trials of sleep health interventions initiated or conducted in school settings and in which behavioral sleep outcomes were measured. Cochrane risk of bias tools were used to assess study quality.

**Results:**

From the 5303 database records and two papers from other sources, 21 studies (22 papers) met the inclusion criteria for this review. These studies involved 10 867 children and adolescents at baseline from 13 countries. Most studies (*n* = 15) were conducted in secondary schools. Sleep education was the most common intervention, either alone (*n* = 13 studies) or combined with other initiatives (stress management training, *n* = 2; bright light therapy, *n* = 1; health education, *n* = 1). Interventions were typically brief in terms of both the intervention period (median = 4 weeks) and exposure (median = 200 minutes). Behavioral outcomes included actigraphy-measured and self-reported sleep patterns, and sleep hygiene. All outcomes had high risk of bias or some concerns with bias. Sleep education interventions were typically ineffective. Later school start times promoted longer sleep duration over 1 week (1 study, high risk of bias).

**Conclusions:**

Current evidence does not provide school-based solutions for improving sleep health, perhaps highlighting a need for complex, multi-component interventions (e.g. whole-of-school approaches) to be trialed.

**Statement of Significance**

Insufficient sleep during childhood and adolescence can contribute to poor health. Many children are not meeting sleep recommendations. School-based interventions have the potential to improve child sleep health. Sleep education interventions have commonly been trialed but have been ineffective, to date, for changing sleep behavior. Delaying school start times seems to result in children sleeping longer, but high-quality studies are needed in this area. The challenges inherent in changing child and adolescent behavior suggest the need for trials of muti-component whole-of-school approaches involving school administrators, educators, parents and guardian, and students.

Insufficient sleep during childhood and adolescence can have adverse consequences for neurocognition (e.g. attention, memory, and intelligence) [1], body composition (e.g. higher adiposity) [1, 2], emotional-behavioral regulation (e.g. behavioral disorders, internalizing/externalizing behaviors) [1, 2], and school-related outcomes (e.g. academic performance [1], daytime sleepiness [3], and tardiness [3]). What counts as optimal sleep, however, varies between individuals and across the lifespan [4]. Public health recommendations are very similar across many jurisdictions, including the United States [5, 6], Canada [7], New Zealand [8], and Australia [9]. The National Sleep Foundation in the United States, for example, recommends 9 to 11 hours of sleep for children aged 6 to 13 years, and 8 to 10 hours for teenagers aged 14 to 17 years [6]. Many children and adolescents are not meeting these recommendations [10, 11]. One US study found that 37% of children aged 6 to 12 years slept less than 9 hours per day, and 31% of adolescents aged 13 to 17 years slept less than 8 hours per day [10]. Studies in other countries, such as Australia [11] and Canada [12], have yielded similar results.

Given the high prevalence of suboptimal sleep, efforts to improve sleep health among children and adolescents are needed. Sleep health refers to “a multidimensional pattern of sleep-wakefulness, adapted to individual, social, and environmental demands, that promotes physical and mental well-being” [13]. Dimensions of sleep health include: duration (“total amount of sleep obtained per 24 hours”), satisfaction/quality (“subjective assessment of *good* or *poor* sleep”), alertness/sleepiness (“ability to maintain attentive wakefulness”), timing (“placement of sleep within the 24-hour day”), and efficiency/continuity (“ease of falling asleep and returning to sleep”) [13].

Universal school-based interventions have the potential to improve population sleep health [14–18]. Research in schools has principally focused on the effects of altering school start times [14–16] and delivering sleep education [16, 18]. Evidence suggests that later school start times may lengthen sleep duration in children [14] and adolescents [14, 15]. In addition, sleep education programs predominantly targeting high school students have had positive effects on sleep knowledge, but mixed effects on sleep outcomes, such as sleep duration, sleep efficiency, sleep onset latency, and time in bed [18]. Research on promoting sleep health among children and adolescents is generally of low quality; however, limiting the certainty with which conclusions can be drawn about intervention effectiveness [15–17]. Identified problems include selection bias, loss to follow-up, unknown or lack of blinding of participants, unknown or low validity and/or reliability of data collection methods, and an absence of information on implementation quality [16]. A recent review (Rigney et al. [18]) of studies on school-based sleep education programs (published up until August 2020); however, showed improvements in the quality of studies over time. These improvements were ascribed to increased use of randomized control trial designs (or, at least, the inclusion of control conditions), larger sample sizes, and greater teacher engagement in delivering sleep education, thus enhancing the external validity of findings [18].

School settings may be highly conducive for interventions to improve sleep health. With children and adolescents spending many of their waking hours in school, these settings would seem to be natural environments for sleep health interventions [18]. School start times and academic workloads are potentially modifiable factors that schools could influence sleep patterns [19]. Furthermore, teachers can be supported through professional development to develop and deliver content on sleep health for their students.

Rigney et al. [18] noted that there was a marked increase in the number of studies published on sleep education programs since 2016. This rise may be indicative of greater attention to school-based interventions for sleep, as well as to sleep health, more broadly. With the increased quality and quantity of research in this area, an up-to-date review is timely to guide research, school and jurisdiction policymaking, and practice. Furthermore, given the focus of Rigney et al. [18] on sleep education programs [18], there is merit in undertaking a broader review to include other types of school-based interventions designed to improve sleep health (e.g. cognitive and behavioral sleep strategies, policies, environmental changes, and multi-component interventions). The purpose of this systematic review is to summarize the effects of school-based interventions on sleep behavior among children and adolescents aged 5 to 18 years.

## Materials and Methods

We used the Preferred Reporting Items for Systematic reviews and Meta-Analyses (PRISMA) 2020 statement [20, 21] to guide the reporting of this systematic review. The review was prospectively registered in PROSPERO (CRD42023429266).

### Selection criteria

Studies were included in this review if they met the following criteria: (1) participants were school children (aged approximately 5 to 18 years) attending regular classes (i.e. mainstream classes rather than special education classes); (2) studies were randomized controlled trials (RCTs); (3) interventions were initiated or conducted in school settings (e.g. education, cognitive and behavioral sleep strategies, policies, environmental changes, and multi-component interventions); (4) interventions included a sleep component designed to promote sleep health; (5) studies included control conditions in which comparison children received education as usual or were exposed to an intervention not designed to promote sleep health; (6) behavioral sleep outcomes were measured, such as those relating to sleep duration (e.g. total night time sleep), sleep–wake circadian patterns (e.g. bedtime, sleep time, and wake time), and sleep hygiene (i.e. engaging in behaviors that promote sleep, such as going to bed at a consistent time, and refraining from behaviors that impede sleep, such as consuming caffeine within 4 hours of going to bed [22, 23]); and (7) papers were published in English in peer-reviewed journals. Studies were excluded if: (1) participation was limited to only include children with sleep difficulties (e.g. insomnia) or health conditions (e.g. anxiety); (2) only non-behavioral sleep outcomes (e.g. sleep knowledge) were measured; or (3) papers were conference abstracts.

### Information sources and search strategy

We identified studies by searching five electronic databases, scanning the reference lists of included papers, and scanning the lists of studies included in previous reviews [3, 14–18, 24]. The electronic databases searched were: Cumulative Index to Nursing and Allied Health Literature Complete, Education Resource Information Center, Education Source, MEDLINE Complete, and PsycINFO (all on the EBSCOHost platform). Search terms were developed using the PICOS (Population, Interventions, Comparators, Outcomes, and Study design) framework [25]. Population search terms for children and adolescents were adapted from a validated search strategy for identifying pediatric studies [26]. Given the review’s focus on school-aged children and adolescents, terms relating to preschool children were removed from the search strategy. Intervention search terms for school-based initiatives were developed based on the search strategies used in a recent systematic review [27] and systematic review protocol [28]. No search terms for comparator conditions were used. Outcome search terms for sleep were developed using terms from a study in which medical subject headings and free text searches were used to retrieve papers on sleep in healthy people [29]. Study design search terms for RCTs were from the Cochrane Highly Sensitive Search Strategy for identifying randomized trials in MEDLINE (sensitivity-maximizing version, 2008 revision) and the Cochrane Cumulative Index to Nursing and Allied Health Literature-Plus filter (the MEDLINE version was adapted for the other databases) [30]. A university librarian reviewed the strategy using the Peer Review of Electronic Search Strategies guideline [31], resulting in minor changes to syntax and search terms. The search strategy was tested and captured all RCTs included in systematic reviews on later school start times [15] and school-based sleep education programs [18]. Appendix A contains the search strategy conducted in MEDLINE (EBSCOHost). No filters were applied to publication language and date. The search was executed on February 01, 2023.

### Study selection

Two reviewers (CJG, CVH) independently assessed the eligibility of each record, with disagreements settled through discussion and, when necessary, consultation with a third reviewer (CS). Records were screened based on title and abstract, and then full text. The study selection process was managed using the Covidence software platform [32].

### Data extraction

Using a data extraction template developed in Microsoft Excel [33], one reviewer (CJG) performed the data extraction, and another (GN) checked the extraction, with disagreements resolved through discussion. The following data were extracted from included studies: citation details (authors and year), country, setting (number of schools, grades, and number of classes), participant characteristics (numbers of children in intervention and control conditions, age ranges, female percentage), study design (randomized control trial and cluster-randomized trial), intervention period (the time span over which the intervention was delivered; e.g. 4 weeks), intervention exposure (the length of the actual delivery of the intervention with children; e.g. four 50 minute classes), intervention description, intervention deliverer, control description, sleep behavior measure types (actigraphy, self-report), follow-up measurement points from baseline (weeks/months), and main findings for behavioral outcomes.

### Risk of bias assessment

Using the revised version of the Cochrane Risk of Bias tool for randomized trials (RoB 2) [34], and an adaptation of the tool for cluster-randomized trials [35], two reviewers (CJG, LDS) independently assessed risk of bias for each outcome of interest in each study. The RoB 2 has five domains: bias arising from the randomization process, bias due to deviations from intended interventions, bias due to missing outcome data, bias in measurement of the outcome, and bias in selection of the reported result. The responses to each domain’s signaling questions are applied using a prescribed algorithm to generate a proposed judgment about the risk of bias for each domain. Another algorithm is then applied to generate the final overall proposed risk of bias judgment for each outcome in a study across all domains, rated as *low*, *some concerns*, or *high* [34]. Disagreements between assessments were resolved through discussion.

### Data synthesis

Our intention was to perform a meta-analysis with the outcome data. Several common issues were identified upon extracting data from the studies. These issues included (1) intervention effects not being provided or being incompletely reported (e.g. effect size (ES)s with no measures of precision); (2) analyses that did not focus on intervention effects directly, but rather on pre-post changes within a given treatment arm or differences between intervention and control conditions at post-intervention only; and (3) non-reporting of descriptive statistics from which ESs could be estimated. Alternative synthesis methods were then considered (e.g. vote counting based on direction of effect, combining *p* values) [36], but could not be implemented for the same reasons. Given these challenges, we drew upon Slaven’s method of *best evidence synthesis* [37], focusing on the risk of bias assessments as a proxy for “best evidence” and ESs (where available) as an adjunct to the presentation of findings. Using this approach, study findings are presented in a narrative synthesis with emphasis on the risk of bias judgments and the evidence (including ESs where available) on the effects of interventions on sleep behavior outcomes in each study.

## Results

**Figure 1.**
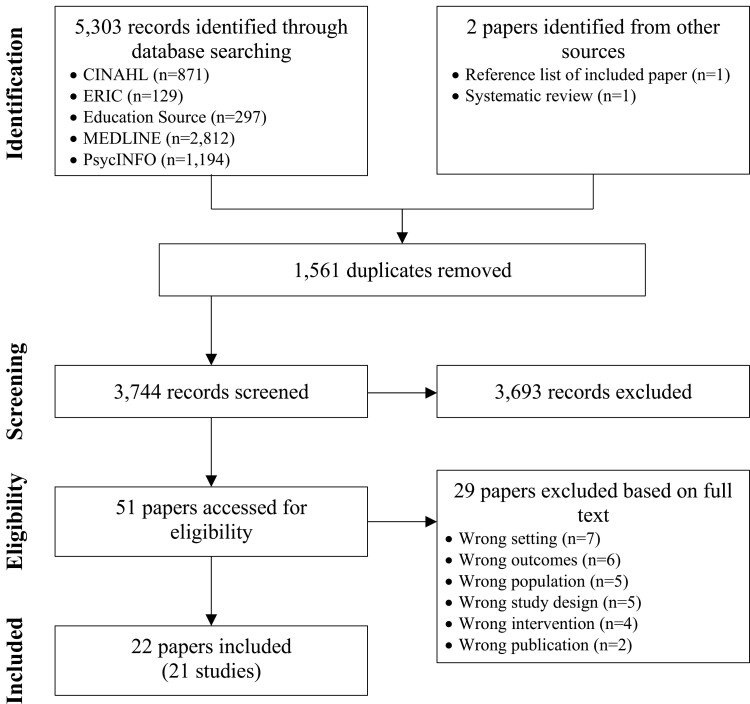
Identification and selection of studies for the systematic review.

Electronic database searches yielded 5303 records, of which 1561 were duplicates (Figure 1). Title and abstract screening followed by full text screening resulted in the identification of 20 eligible papers [38–57]. Two additional papers were identified separately from the reference list of an included paper [58] and a previous systematic review [59]. Two other included papers presented duplicate results [46, 59]. Therefore, in total, 22 papers [38–58] with results from 21 studies were included in this review.

### Study characteristics

The studies were conducted across 13 countries (Table 1). Participants at baseline were 10 867 children and adolescents, with the number of students recruited in each study ranging from 21 [58] to 3713 [57] (median = 148, interquartile range [IQR]: 58–352). The studies took place in elementary/primary schools (ages 5 to 10 years, *n* = 3 studies) [39, 52, 54], junior high/middle schools (ages 11 to 13 years, *n* = 3 studies) [50, 53, 56], and secondary/high schools (ages 14–18 years; *n* = 15 studies, 16 papers) [38, 40–49, 51, 55, 57–59]. Almost all studies were cluster-randomized trials (*n* = 19 studies) [38–45, 47–50, 52–58], with the remaining two studies (with results presented in three papers) being parallel RCTs [46, 51, 59].

**Table 1.** Characteristics of the Studies and Summary of Findings

Study and country	Setting and participants	Design	Intervention Condition and period (exposure)	Control condition	Measure type and follow-up points	Findings			
						Timing	Efficiency	Duration	Hygiene
Baldursdottir et al. (2017) [38] Iceland	Four first-year classes from four upper-secondary schools Int: *n* = 26, 15–16 years, 56% female Con: *n* = 27, 15–16 years, 65% female	CRT	Physical activity 3 weeks (-)	No tracking of steps nor text messages during the intervention period.	Self-report 3 weeks		✓/NS		
Bavarian et al. (2016) [39] United States	Fourteen kindergarten-to-grade-six and kindergarten-to-grade-eight schools Int: *n* = 295, M = 8.3 years (*SD* = 0.58), 53% female Con: *n* = 299, M = 8.3 years (*SD* = 0.55), 55% female	CRT	Personal development 6 years (-)	School as usual	Self-report 6 years	NS			
Beijamini & Louzada (2012) [58] Brazil	Two classes in 1 school Int: *n* = 10, 13–14 years old, 70% female Con: *n* = 11, 13–14 years old, 36% female	CRT	Sleep education 4 days (200 min)	Classes as usual	Actigraphy 5 days	NS	NS	NS	
Bonnar et al. (2015) [40] Australia	Two year 11 classes from each of six high schools *n* = 193, M = 16.3 ± 0.4 years, 79% female	CRT	Sleep education and bright light therapy 4 weeks (-)	Classes as usual	Self-report 5 weeks, 11 weeks	NS	✓/NS	✓	
Cain et al. (2011) [41] Australia	Two year 11 classes from each of three high schools *n* = 104 (intervention = 53, control = 51), M = 16.2 ± 0.4 years, 60% female	CRT	Sleep education 4 weeks (200 min)	Classes as usual	Self-report 6 weeks, 12 weeks	NS	NS	NS	
Das-Friebel et al. (2019) [42] Switzerland	Thirty-four seventh- to twelfth-grade classes from seven schools Int: *n* = 192, M = 15.0 years (*SD* = 1.71), 43% female Con: *n* = 160, M = 15.3 years (*SD* = 1.56), 50% female	CRT	Sleep education 25 min (25 min)	Placebo control	Self-report 4 weeks			NS	✓/NS
Inhulsen et al. (2022) [43] The Netherlands	Fifty-nine second- and third-grade classes from 10 high schools Int: *n* = 605, M = 13.4 years (*SD* = 0.76), 59% female Con: *n* = 367, M = 13.2 years (*SD* = 0.64), 47% female	CRT	Sleep education 1.5 weeks (135 min)	Wait-list control	Self-report 1.5 weeks, 3 months			×/NS	NS
John et al. (2016) [44] India	Sixth to twelfth grades from two schools Int: *n* = 34, 53% female, [M = 14 years (*SD* = 2.15) across intervention and control] Con: *n* = 24, 46% female	CRT	Stress management and sleep education 2 weeks (117.5 min)	School as usual	Self-report 14 days, 6 weeks			NR	NS
John et al. (2017) [45] India	Sixth to twelfth grades from 6 schools *n* = 660, M = 13.6 years (*SD* = 1.70), 50% female	CRT	Stress management and sleep education 2 weeks (112.5 min)	Wait-list control	Self-report 2 weeks, 6 weeks		NR	NR	NR
Kira et al. (2014) [46] and Blunden et al. (2012) [59] New Zealand	Two classes (year 9 and year 11/12) from 1 high school Int: *n* = 15, M = 14.8 years (*SD* = 1.1), 47% female Con: *n* = 14, M = 14.7 years (*SD* = 1.2), 43% female	RCT	Sleep education 5 weeks (200 min)	Wait-list control (condensed version of the intervention)	Self-report 5 weeks, 10 weeks	✓/NS		✓/NS	NS
Lin et al. (2018) [47] Iran	Two classes from each of 48 schools Int: *n* = 1425, M = 15.5 years (*SD* = 1.08), 55% female Con: *n* = 1416, M = 15.12 years (*SD* = 1.50), 52% female	CRT	Sleep education 2 months (300 min)	Wait-list control	Self-report 3 months, 8 months			✓/NS	
Lufi et al. (2011) [48] Israel	Eighth grade from one school *n* = 47, M = 13.8 years (*SD* = 0.28), 57% female	CRT	School start times 1 week (-)	Regular school starting time	Actigraphy 1 week, 2 weeks	✓/NS	NS	✓	
Moseley et al. (2009) [49] Australia	Two year 11 psychology classes from each of two secondary schools *n* = 81, M = 15.6 years (*SD* = 0.60), 67% female	CRT	Health education 4 weeks (200 min)	Classes as usual	Self-report 4 weeks, 6 weeks		NS	NS	
Rigney et al. (2015) [50] Australia	Year 6/7 classes from 12 junior schools (each school provided ≥ 1 class) *n* = 296, M = 12.2 years (*SD* = 0.60), 59% female	CRT	Sleep education 4 weeks (200 min)	Classes as usual	Actigraphy, self-report 6 weeks, 18 weeks	✓/NS	NS	✓/NS	NS
Sousa et al. (2013) [51] Brazil	Twelfth grade from one school *n* = 34, M = 16.8 years (*SD* = 0.60), 84% female	RCT	Sleep education 5 days (225 min)	Classes as usual	Self-report 3 weeks	NR		NR	
Tamura & Tanaka (2014) [52] Japan	Two classes (years 4, 5, and 6) from each of 2 elementary schools Int: *n* = 72, ages not reported, 44% female Con: *n* = 76, ages not reported, 49% female	CRT	Sleep education 45min (45 min)	Classes as usual	Self-report 2 weeks	✓/NS		✓/NS	NR
Tamura & Tanaka (2016) [53] Japan	Eight seventh grade classes from 5 junior high schools Int: *n* = 122, aged 12-13 years, 42% female Con: *n* = 121, aged 12-13 years, 58% female	CRT	Sleep education 50 min (50 min)	Wait-list control	Self-report 2 weeks	✓/NS	✓/NR	✓	✓
Uhlig et al. (2019) [54] The Netherlands	Five eighth grade classes in a primary school Int: *n* = 52, M = 10.1 years (*SD* = 1.23), % female not reported Con: *n* = 23, M = 10.4 years (*SD* = 0.78), % female not reported	CRT	Music education 4 months (720 min)	Wait-list control	Actigraphy 4 months		NS	✓	
van Rijn et al. (2020) [55] Singapore	Twelve eighth grade classes in a secondary school Int: *n* = 102, M = 14.0 years (*SD* = 0.37), 0% female Con: *n* = 108, M = 14.0 years (*SD* = 0.27), 0% female	CRT	Sleep education 5 weeks (240 min)	Placebo control	Actigraphy 6 weeks, 11 weeks	NS	✓/NS	NS	
Wing et al. (2015) [57] Hong Kong	Seventh to eleventh grades from 14 secondary schools Int: *n* = 1545, M = 14.9 years (*SD* = 0.11), 70% female Con: *n* = 2168, M = 14.6 years (*SD* = 0.18), 52% female	CRT	Sleep education ~3 months (80 min)	School as usual	Self-report ~4 months	✓/NS		✓/NS	NS/NR
Wolfson et al. (2015) [56] United States	Twelve seventh grade classes in 2 middle schools Int: *n* = 70, M = 12.5 years (*SD* = 0.56), 60% female Con: *n* = 73, M = 12.6 years (*SD* = 0.48), 59% female	CRT	Sleep education 4 weeks (320 min)	Placebo control	Self-report ~5 weeks, ~10-11 months, ~15-16 months	✓/NS		✓/NS	✓/NS

CRT, cluster-randomized trial; RCT, parallel randomized controlled trial. Timing, the location of sleep within a 24-hour period (variables include consistent/typical bedtime, lights-out time, sleep onset/offset times, wake-up time). Efficiency, the proportion of time asleep of the time dedicated for sleep (variables include sleep efficiency, sleep onset latency, nightly awakenings, sleep episode length, snooze time). Duration, the amount of time asleep within a 24-hour period (variables include sleep duration, total sleep time, 24-hour sleep, time in bed, and discrepancy between school day and weekend out-of-bed times). Hygiene, behaviors and environmental factors that promote sleep (variables include sleep hygiene, caffeine consumption, alcohol consumption, bedtime routine, and bedtime electronic media use). ✓, significant effects favoring intervention condition. ×, significant effects favoring control condition. NS, no significant difference between conditions. NR, not reported. ✓/NS, NS/NR, and ×/NS, multiple results with inconsistent outcomes (e.g. a significant effect at an initial follow-up point, but a null result at a subsequent follow-up point).

Most interventions were sleep education alone (*n* = 13 studies, 14 papers) [41–43, 46, 47, 50–53, 55–59] or sleep education in combination with stress management training (*n* = 2 studies) [44, 45], bright light therapy (*n* = 1 study) [40], or health education (*n* = 1 study) [49]. The remaining interventions involved music education [54], personal development [39], physical activity [38], and delayed school start times [48] (*n* = 1 study each, respectively). Details of the interventions are provided in Supplementary Table S1.

Intervention periods (i.e. time spans over which interventions were delivered) ranged from 25 minutes [42] to 6 years [39] (median = 4 weeks, IQR: 1–5). The intervention exposure (i.e. total length of actual delivery of the intervention to which children were exposed; e.g. two 60-minute sessions equals 120 minutes exposure) were available for 17 studies (18 papers) [41–47, 49–59]. Intervention exposures ranged from 25 [42] to 720 [54] minutes (median = 200 minutes; IQR: 113–225). In one study with an intervention period of 6 years, children in each grade from kindergarten through to sixth grade received 140 personal development sessions (with each session lasting 15–25 minutes), and grades seven and eight received 70 sessions (each lasting 20 minutes); sleep was incorporated into one of the six units taught [39]. The studies in which intervention exposures could not be ascertained included those where (1) adolescents monitored their physical activity and sleep and received short motivational messages to increase their daily step count [38]; (2) students received four, weekly 50 minutes sleep education classes, with or without parental involvement, and bright light (i.e. although the duration of the sleep classes is reported, the duration of the bright light component of the intervention was unreported) [40]; or (3) school start times were delayed by 1 hour [48].

Researchers (*n* = 8 studies) [38, 40–42, 45, 49, 51, 53] and teachers or other school staff (*n* = 6 studies, 7 papers) [39, 43, 46, 48, 50, 55, 59] delivered most interventions. Others who delivered the interventions were facilitators (*n* = 2 studies) [47, 56], music therapists (*n* = 1 study) [54], physicians (*n* = 1 study) [57], and a sleep instructor (*n* = 1 study) [52]. The intervention deliverers were not reported in two studies [44, 58].

Control conditions were no treatment (e.g. classes as usual; *n* = 13 studies) [38–42, 44, 48–52, 57, 58], wait-list control (*n* = 6 studies, 7 papers) [43, 45–47, 53, 54, 59], and placebo control (*n* = 2 studies) [55, 56]. The placebo controls were a healthy living program (with no sleep-related content) [55] and group-based and telephone contact focused on observing and reporting sleep patterns (with no content on ways to improve sleep habits) [56].

Sleep behaviors were measured using actigraphy in five studies [48, 50, 54, 55, 58] and self-reported in 17 studies (18 papers) [38–47, 49–53, 56, 57, 59]. The initial follow-up measurement point from baseline ranged between 5 days and 6 years (median = 4 weeks, IQR:2–6 weeks). These initial follow-up points were at the end of the interventions (9 studies, 10 papers [38, 39, 43–46, 48, 49, 54, 59]), within a week of the intervention ending (2 studies [40, 58]), or 1 week (2 studies [55, 56]), 2 weeks (5 studies [8, 41, 50, 52, 53]), or 4 weeks (3 studies [42, 47, 57]) after the interventions ended. Twelve studies (13 papers) [40, 41, 43–50, 55, 56, 59] had second follow-up points ranging from 2 weeks [48] to approximately 10–11 months [56] (median = 11 weeks, IQR:6–14 weeks). These second follow-up points were 1 week (1 study [48]), 2 weeks (1 study [49]), 3 weeks (1 study [44]), 4 weeks (1 study [45]), 5 weeks (2 studies, 3 papers [40, 46, 59]), 6 weeks (1 study [55]), 8 weeks (1 study [41]), 3 months (1 study [43]), 14 weeks (1 study [50]), 6 months (1 study [47]), or approximately 9–10 months (1 study [56]) after the interventions ended. One study had a third follow-up point at approximately 15–16 months (approximately 14–15 months after the intervention ended) [56].

### Risk of bias

All outcomes in all studies received overall ratings of high risk of bias or some concerns with bias (Supplementary Table S2). These ratings were primarily due to risk-of-bias judgments for Domains 1/1a, 1b, 4, and 5. The domain 1/1a ratings of high risk of bias or some concerns with bias were mainly due to the lack of information about whether allocation sequences were concealed (*n* = 14 studies [38–42, 44, 45, 48, 49, 51, 53, 55, 56, 58]) or to the nonuse of concealment (*n* = 1 study [57]). Similarly, the domain 1b ratings were primarily due to the lack of information as to whether participants were identified and recruited before the randomization of clusters (*n* = 10 studies [42–45, 48, 49, 52, 53, 57, 58]) or to evidence that participants were not recruited prior to randomization (*n* = 3 studies [40, 41, 56]). Domain 4 assessments of some concerns with bias were due to the possibility that knowledge of the intervention received could have influenced participants responses to self-report measures (*n* = 16 studies, 17 papers [38–47, 49, 51–53, 56, 57, 59]). Domain 5 ratings of some concerns were due to unknown analysis intentions (e.g. due to the unavailability of trial protocols or registrations; *n* = 19 studies [38–53, 56–58]).

### Effects of interventions on behavioral outcomes

The effects of interventions on behavioral outcomes are summarized in Table 2 (sleep patterns) and Table 3 (sleep hygiene), with outcomes for each study provided in Table 1 and, in greater detail, in Supplementary Table S1. The behavioral outcomes assessed in the studies were actigraphy-measured sleep patterns, self-reported sleep patterns, and sleep hygiene.

**Table 2.**
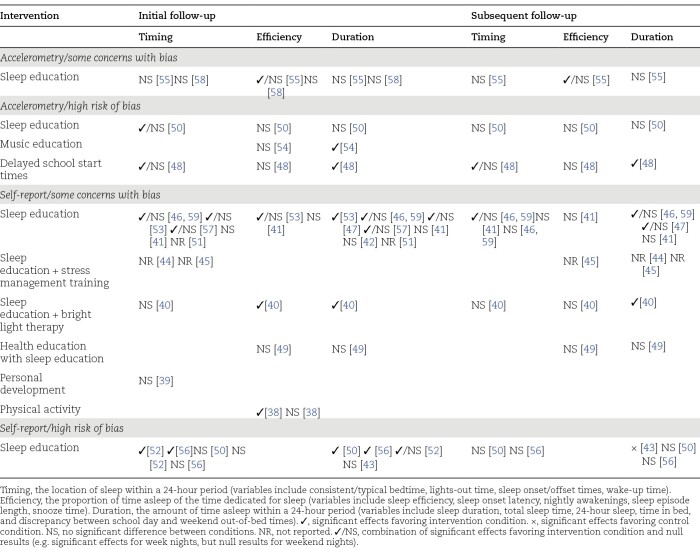
Summary of the Effects of the Interventions on Sleep Patterns by Outcomes and Level of Bias

**Table 3.**
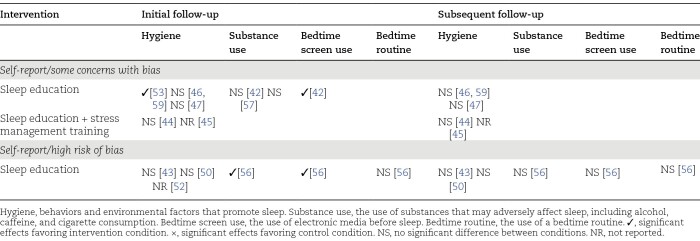
Summary of the Effects of the Interventions on Sleep Hygiene by Level of Bias

#### Effects of interventions on actigraphy-measured sleep patterns.

Sleep education interventions alone were investigated in three studies in which actigraphy-measured sleep patterns were assessed, with two having some concerns with bias [55, 58] and one having high risk of bias [50]. In the two studies that have some concerns with bias, sleep education alone had no significant effects on sleep patterns [55, 58]. Another study investigating sleep education alone, but which had high risk of bias, showed a significant effect on wake time favoring the intervention condition at 6 weeks (following the intervention), but not at 18 weeks [50]. No significant intervention effects on other sleep parameters were found.

A music education intervention was trialed in one study with high risk of bias [54]. The intervention had a significant effect on sleep duration at 4 months favoring the intervention condition, but not on other sleep outcomes.

Delaying school start times by 1 hour for 1 week was trialed in one study with high risk of bias [48]. The intervention had substantial favorable, and statistically significant, effects on sleep offset time (later sleep offset; partial η^2^ = 0.71) and sleep duration (partial η^2^ = 0.29), but no significant effects on sleep onset time or sleep efficiency.

#### Effects of interventions on self-reported sleep patterns.

Sleep education interventions alone were trialed in 11 studies (12 papers) in which self-reported sleep patterns were measured, with seven studies (eight papers) having some concerns with bias [41, 42, 46, 47, 51, 53, 57, 59] and four having high risk of bias [43, 50, 52, 56]. Of the seven studies (eight papers) that have some concerns with bias, significant intervention effects favoring the intervention condition on at least one sleep outcome were reported at the initial follow-up points in four studies (five papers) [46, 47, 53, 57, 59], no significant intervention effects were found in two studies [41, 42], and intervention effects were not reported in one study [51]. Three studies (four papers) had additional follow-up points (10 weeks [46, 59],12 weeks [41], and 8 months [47]), with the findings from two studies (three papers) [46, 47, 59] showing favorable, and statistically significant, interventions effects for sleep duration on weekend nights (but not week nights), and no significant effects in one study [41]. Of the four studies with high risk of bias, three studies showed significant effects favoring the intervention conditions on at least one outcome at initial follow-up points [50, 52, 56]. Three of these studies had subsequent follow-up points (18 weeks [50], 3 months [43], and approximately 10–11 months and approximately 15–16 months [56]), with one study showing a significant intervention effect for sleep duration favoring the control condition [43], and the other two studies showing no significant intervention effects [50, 56].

Sleep education was trialed in combination with stress management training in two studies [44, 45], with bright light therapy in one study [40], and as part of health education in one study [49]. All studies had some concerns with bias. For the two studies on stress management and sleep education, no intervention effects were reported [44, 45]. Bright light therapy in conjunction with sleep education had favorable, and statistically significant, effects on sleep onset latency post-intervention (5 weeks), but not at 11 weeks, and on total sleep time on school nights at both follow-up points [40]. Sleep education, as a component of health education, had no significant effects on sleep onset latency and total sleep time [49].

Personal development was investigated in one study that has some concerns with bias [39]. The intervention had no significant effect on consistent bedtimes by 9 pm on school nights.

Physical activity was trialed in one study that has some concerns with bias [38]. A favorable, and statistically significant, effect on sleep onset latency at the end of the intervention was found. There was no significant intervention effect on nightly awakenings, however.

#### Effects of interventions on sleep hygiene.

Sleep education interventions alone were trialed in six studies (seven papers) in which sleep hygiene was assessed, with three studies (four papers) having some concerns with bias [46, 47, 53, 59] and three having high risk of bias [43, 50, 52]. Of the three studies that have some concerns with bias, favorable, and statistically significant, intervention effects were reported in one study [53] and no significant intervention effects were found in two studies (three papers) [46, 47, 59]. Two studies each had two follow-up points, with no significant intervention effects observed at either follow-up point [46, 47, 59]. Similarly, among the studies with high risk of bias, no significant intervention effects were found in three studies [43, 50, 56], and intervention effects were unreported in one study [52].

Stress management combined with sleep education was investigated in two studies that had some concerns with bias [44, 45]. No significant intervention effects were found in one study [44] and intervention effects were unreported in the other study [45].

Sleep education alone was trialed in one study in which bedtime routine (an aspect of sleep hygiene) was assessed, with this study having high risk of bias [56]. No significant intervention effects were found at either of the two follow-up points in the study.

Sleep education interventions alone were investigated in two studies in which bedtime screen use (an aspect of sleep hygiene) was measured, with one having high risk of bias [56] and the other having some concerns with bias [42]. In the study with some concerns, a significant effect favoring the intervention condition was found at 4 weeks [42]. For the study with high risk of bias, a significant effect favoring the intervention condition was found at approximately 5 weeks but not at approximately 10–11 months nor at approximately 15–16 months [56].

Sleep education interventions alone were trialed in three studies in which substance use (an aspect of sleep hygiene) was assessed, with one having high risk of bias [56] and two having some concerns with bias [42, 57]. Neither of the studies with some concerns demonstrated significant intervention effects for caffeine [42, 57] or alcohol consumption [42, 57], or cigarette smoking [57]. In the study with high risk of bias, a significant effect on PM caffeine use favoring the intervention condition was found at approximately 5 weeks but not at approximately 10–11 months nor at approximately 15–16 months [56].

## Discussion

The studies included in this review generated findings that suggest that short-term school-based education interventions are associated with minimal to no changes in the sleep behaviors of adolescents. Across studies, the outcomes had high risk of bias or some concerns with bias, and the effects of the interventions on behavior were, at best, transient, with minimal evidence of effects beyond the completion of the interventions. That is, for those studies with multiple follow-up points, intervention effects demonstrated post-intervention were not typically sustained at subsequent follow-up points. Fewer studies have been conducted with children than adolescents, with outcomes from these studies often having high risk of bias and producing equivocal results.

At the initial (and, in some studies, only) follow-up points, sleep education alone interventions were effective for changing sleep patterns in 9 of the 14 studies and sleep hygiene in three of the nine studies. In the studies that generated statistically significant results, improvements were typically seen for a minority of the sleep variables measured (e.g. wake-up time, but not bedtime, sleep onset latency, sleep duration or sleep efficiency [50]) or for only certain nights of the week (e.g. weekend nights not weeknights [55]). When there were subsequent follow-up points, sleep education alone was effective for changing sleep patterns in three of the nine studies and sleep hygiene in zero of the five studies. These findings align with the broader evidence on promoting child and adolescent health, which shows that the effects of classroom-based health education on behavior are inconsistent and short-term [60–63]. Like health education more generally [60, 63], sleep education increases knowledge [18], but this increased knowledge does not appear to translate into lasting behavior change (i.e. weeks or months after interventions cease) as evidenced in the findings from the included studies. In contrast, evidence suggests that the World Health Organization’s holistic Health Promoting Schools framework [64] and whole-of-school approaches [63] can be effective for promoting student health. The Health Promoting Schools framework was developed in response to the limited success of traditional health education for improving health outcomes, and typically incorporates three characteristics: (1) promotion of health throughout the formal curriculum, (2) promotion of health via the informal curriculum (e.g. values and attitudes espoused within schools, and the physical environments of schools), and (3) engagement with families, outside agencies, and wider communities in recognition of their importance in influencing child health [64]. Such initiatives have positive, generally small, effects on body mass index, fruit and vegetable intake, physical activity, physical fitness, tobacco use, and bullying [64]. Whole-of-school approaches are multi-component school-based interventions incorporating, for example, school policy changes, parental involvement, and work with communities [63]. These initiatives have been effective for preventing bullying and smoking, and for promoting sexual health [63]. Drawing on the evidence on what has shown to be effective for promoting health in schools, developing sleep health interventions using whole-of-school approaches may be a worthwhile way forward.

One component of whole-of-school approaches to improving sleep health may be later school start times. Evidence from one study with high risk of bias indicates that delaying school start times by an hour resulted in children sleeping longer after a week [48]. The findings from this trial are consistent with those of two recent reviews [14, 15]. In a systematic review, Marx et al. [15] combined the effects from three non-randomized cross-over trials, which showed that students at schools with later start times slept longer than those with earlier start times (mean difference = 1.39 hours; 95% confidence interval [CI]: 0.38, 2.39). An additional six studies that could not be included in the meta-analysis (one of which was a CRT included in the present review [48]) also showed that later school start times were positively associated with sleep duration [15]. These reviewers rated the quality of evidence as generally very low and noted that there were challenges with conducting studies in this area (e.g. schools are typically unwilling or unable to give researchers control over scheduling and data collection that would enable RCTs to be undertaken) [15]. In subsequent meta-analysis, delayed school start times were associated with longer sleep duration (ES = 0.109; 95% CI: 0.019, 0.199; 23 studies) but not bedtimes (ES = 0.101; 95% CI: −0.048, 0.250; 17 studies) or wake times (ES = 0.021; 95% CI: −0.236, 0.278; 15 studies) [14]. Given the inherent challenges of conducting research in school environments [15, 65, 66], researchers wishing to conduct experimental studies will likely need to work with jurisdictional education departments and convenience samples of schools that have an interest in modifying start times.

Although the rapid growth in sleep education programs was noted in a prior review [18], we only identified one additional trial published since that review that tested a sleep education intervention [43]. That intervention incorporated three 45 minutes classroom sessions with interactive assignments and an educational website. No intervention effect on sleep duration or sleep hygiene was observed at 1.5 weeks, and an effect on sleep duration of 22 minutes per night in favor of the control condition was apparent at 3 months. The researchers attributed this unexpected result to low adherence with completing the sleep diary. This finding adds to the broader body of evidence observed in our review that suggests that sleep education in isolation has no effect on sleep behavior.

Although it is accepted that conducting research in school settings is challenging [65, 66], and an improvement in the quality of studies was noted in a previous review [18], all trials were rated as having a high risk of bias or some concerns with bias in our review. Key areas of concern were (1) limited or no details about recruitment, randomization, and analysis intentions, and (2) the use of self-report measures in the context of trials where participants probably had knowledge of the interventions they received. With regards to the first issue, it is unclear whether the lack of information about recruitment and randomization is indicative of deviations from recommended practice or inadequate reporting. Similarly, with the non-disclosure of analysis intentions, it is uncertain whether results were selectively reported. Publishing trial protocols, including more details in trial registries, and adhering to the consolidated standards of reporting trials (CONSORT) statements [67–69] would enable better assessments of the quality of trials in this space. With respect to the second issue, and acknowledging that the blinding of participants in these types of trials is challenging, there seems to be merit in using objective measures of sleep patterns.

Strengths of this systematic review include its focus on RCTs and the breadth of interventions eligible for inclusion. RCTs are arguably the most reliable method for testing interventions for promoting sleep health. An emphasis on RCTs serves to elevate research using rigorous forms of evaluation. Unlike previous reviews that have separately focused on sleep education [18, 24] and later school start times [14, 15], this review draws together different interventions in a single analysis.

This systematic review has limitations. First, we were unable to undertake a meta-analysis as originally planned. The quality of statistical analysis and reporting in the included studies was such that calculating ESs for outcomes across studies was not feasible. Also, a diverse set of outcomes were measured, which would have complicated attempts to conduct a meta-analysis. Contacting researchers for further information on their studies was not undertaken due to resource constraints and the expected improbability of obtaining sufficient information to make the exercise worthwhile. The approach used—best evidence synthesis—would have benefitted from the availability or calculation of ESs for more statistics than could be extracted from studies included in this review. Second, the inconsistent operationalization of key sleep outcomes across trials hampered the synthesis of study findings. We encourage work to standardize how sleep patterns and sleep hygiene are measured in trials of school-based interventions for children and adolescents. Third, with marked differences between children and adolescents, the broad range of ages included in this review (5 to 18 years) could be an issue. Only 3 of the 21 trials, however, included children from 5 to 10 years, meaning that separate age-related analyses would not have been productive. Further research is needed to establish (1) the efficacy of sleep health interventions on child sleep behaviors (2) whether there may be an optimal age of intervention, and (3) how interventions may need to change as children and adolescents grow older.

The research reviewed shows that efforts to promote longer sleep duration, largely through using education alone to encourage earlier bedtimes and better sleep hygiene in adolescents, are probably ineffective. The best that can be hoped for, it seems, is short-term improvements in sleep duration and hygiene. Findings from this review highlight a need to move away from education-only interventions for changing sleep behavior. Multi-component whole-of-school approaches—involving school administrators, educators, parents, and students among others—may hold more promise and should be trialed.

## Supplementary Material

zpae019_suppl_Supplementary_Tables_S1-S2

## Data Availability

No new data were generated or analyzed in support of this research.

## Appendix A

MEDLINE

MEDLINE (1865 to present) was searched via EBSCOHost.

((MH “Pediatrics” OR MH “Child” OR MH “Adolescent” OR MH “Minor”) OR minor* OR boy# OR boyfriend OR boyhood OR girl* OR kid OR kids OR child* OR schoolchild* OR adolescen* OR juvenil* OR youth* OR teen* OR under*age* OR pubescen* OR p#ediatric* OR “young person” OR “young people”)

AND

((MH “Schools”) OR AB (school* OR education OR lesson*) OR TI (school* OR education OR lesson*))

AND

((MH “Sleep+”) OR (MH “Sleep Wake Disorders+”) OR AB sleep OR TI sleep OR (sleep N3 (efficiency OR latency OR stages OR maintenance OR onset OR total OR time OR duration OR satisfaction OR quality OR behavio#r* OR length OR depriv* OR sufficient OR insufficient OR short OR hygiene)) OR “rapid eye movement” OR REM OR sleepiness OR tiredness OR “night waking” OR “sleep-wake cycle” OR bedtime OR (bed N3 time))

AND

((PT “randomized controlled trial” OR PT “controlled clinical trial” OR AB (randomized OR randomized OR placebo OR randomly OR trial OR groups)) NOT (MH “Animals+” NOT MH “Humans”)))
