# Supplementary material for: Sleep behavioral outcomes of school-based interventions for promoting sleep health in children and adolescents aged 5 to 18 years: a systematic review
Source: Sleep Adv. 2024 Mar 29;5(1):zpae019. doi: 10.1093/sleepadvances/zpae019 (PMC10996385; doi:10.1093/sleepadvances/zpae019)
Supplement: zpae019_suppl_Supplementary_Tables_S1-S2 [file zpae019_suppl_supplementary_tables_s1-s2.pdf]

Sleep behavioural outcomes of school-based interventions for promoting sleep health in children and adolescents aged 5 to 18 years: A systematic review

Cadeyrn J. Gaskin, Deakin University, Geelong, Australia

Carolina Venegas Hargous, Deakin University, Geelong, Australia

Lena D. Stephens, Deakin University, Geelong, Australia

Gunchmaa Nyam, Deakin University, Geelong, Australia

Victoria Brown, Deakin University, Geelong, Australia

Natalie Lander, Deakin University, Geelong, Australia

Serene Yoong, Deakin University, Geelong, Australia

Bridget Morrissey, Deakin University, Geelong, Australia

Steven Allender, Deakin University, Geelong, Australia

Claudia Strugnell, Deakin University, Geelong, Australia

Supplementary Material

Supplementary Table 1: Intervention details and main findings for the behavioural outcomes.

Supplementary Table 2: Risk of bias.

Supplementary Table 1: Intervention details and main findings for the behavioural outcomes.

Study and Country	Intervention Description	Intervention Deliverer	Main findings for Behavioural Outcomes
Baldursdottir et al. (2017)[38]<br><br>Iceland	Adolescents wore unsealed pedometers, received a short motivational message to aim for a minimum of 8,000-10,000 steps per day, completed sleep diaries, and received text messages prompting them to track their steps, maintain their sleep diaries, and wear the pedometers.	Researchers	**Sleep onset latency:** Significant intervention effect favouring intervention group, F(1, 44)=4.90, p=.03, partial η^2=0.10<br>**Nightly awakenings:** No intervention effect
Bavarian et al. (2016)[39]<br><br>United States	Positive Action - a social-emotional and character development program, of which the kindergarten through eighth grade portion of the curriculum was implemented, consisting of 140 15-20 min, age-appropriate, interactive lessons per grade, which were taught 4 days per week to grades K-6, and 70 20-min lessons per grade which were taught 2 days per week to grades 7 and 8. Sleep is addressed in one of the six units.	Teachers and other school staff (trained in Positive Action and given the flexibility to adapt lessons; e.g., changing names of story characters to be culturally appropriate)	**Consistent bedtime (by 9pm on school nights):** No significant intervention effect (B=0.09; 95% CI: -0.05, 0.22; Hedges g=0.35)
Beijamini & Louzada (2012)[58]<br><br>Brazil	Four 50min classes on consecutive days covering sleep and health, sleep functions, the importance of sleep, sleep and adolescence, and sleep hygiene.	Not reported	**Sleep onset time**: No significant intervention effects for school nights (p=.49) or weekend nights (p=.56)<br>**Sleep offset time:** No significant intervention effects for school nights (p=.53) or weekend nights (p=.40)<br>**Sleep duration:** No significant intervention effects for school nights (p=.22) or weekend nights (p=.87)<br>**Sleep efficiency:** No significant intervention effects for school nights (p=.25) or weekend nights (p=.81)
Bonnar et al. (2015)[40]<br><br>Australia	(1) Sleep education program (SEP; four, weekly, 50min sleep education classes fitted within a motivational interviewing framework) + PI (parental involvement; parents could access four, 2-4min YouTube videos with content coinciding with information being taught in the SEP) + on the third weekend students were instructed to incrementally advance their wake-up times by 30mins, but stay in dim light for 30-60mins.<br>(2) SEP + BL (bright light; students using portable light devices from the third weekend to incrementally advance	Researchers (qualified school teachers who were also registered psychologists trained in motivational interviewing)	**Bedtime on school nights:** No significant intervention effect, F(1,11)=1.75, p=.065<br>**Sleep onset latency:** Significant intervention effect at 5 weeks with intervention groups, on average, improving 14.6 minutes and the control condition improving 5.9 minutes, F(1,11)= 1.76, p<.0001, partial η^2=0.10; no significant effect at 11 weeks<br>**Total sleep time on school nights:** Significant intervention effect favouring intervention groups, F(1,11)=5.68, p<.0001

	their wake-up time by 30 mins then use these devices for 30-60mins.<br>(3) SEP + BL (from the third weekend) + PI.		
Cain et al. (2011)[41]<br><br>Australia	Four, weekly, 50min sleep education classes fitted within a motivational interviewing framework.	Researcher (a qualified school teachers who was also a registered psychologist trained in motivational interviewing)	**Typical bedtime:** No significant intervention effect<br>**Lights-out time:** No significant intervention effect<br>**Sleep onset latency:** No significant intervention effect<br>**Total sleep time:** No significant intervention effect<br>**Wake-up time:** No significant intervention effect<br>**Discrepancy between school day and weekend out-of-bed times:** No significant intervention effect
Das-Friebel et al. (2019)[42]<br><br>Switzerland	Psychoeducation for students (with an emphasis on avoiding electronic media use at night and evening-time caffeine consumption) and information for parents.	Trained researchers	**Sleep duration:** No significant intervention effects for week days (β=0.00; 95% CI: -0.15, 0.16; p=.950) and weekend days (β=0.08; 95% CI -0.16, 0.31; p=.521)<br>**Caffeine consumption:** No significant intervention effects (β=0.06; 95% CI: -0.16, 0.27; p=.610)<br>**Bedtime electronic media use:** Significant intervention effect favouring intervention group (β=-0.87; 95% CI: -1.43, -0.32; p=.002)
Inhulsen et al. (2022)[43]<br><br>The Netherlands	Charge Your Brainzzz - three 45min classroom sessions with interactive assignments and an educational website. Accompanying homework included a serious game and an assignment for students and parents.	Biology teachers or mentors of the classes	**Sleep duration:** No significant intervention effect at 1.5 weeks (B=0.90; 95% CI: -7.59, 9.40) and a significant intervention effect of 22 minutes per night favouring control condition at 3 months (B=-22.29; 95% CI -32.81, -11.76)<br>**Sleep hygiene:** No significant intervention effects at 1.5 weeks (B=-0.07; 95% CI: -0.17, 0.03) and 3 months (B=0.06; 95% CI: -0.05, 0.16)
John et al. (2016)[44]<br><br>India	Sleep promotion program with three components: (a) a sleep hygiene education session (50min), (b) visualisation and imagery training for stress reduction and relaxation (one 25min video and another 25-30min video), and (c) tips on time management (15min session). Students were asked to practice visualisation for 5-10mins every day before sleep.	Not reported	**Sleep duration:** Intervention effects unreported<br>**Sleep hygiene:** No significant intervention effects
John et al. (2017)[45]<br><br>India	Sleep promotion program with three components: (a) education module on sleep hygiene (split into 35min and 15min sessions), (b) video-based imagery training for stress reduction and relaxation (two 20–25-min sessions over 2 days), and (c) education session on time management skills (15–20min) along with a time planner.	Researcher	**Sleep onset latency:** Intervention effects unreported<br>**Sleep duration:** Intervention effects unreported<br>**Sleep hygiene:** Intervention effects unreported

Kira et al. (2014)[46] and Blunden et al. (2012)[59]<br><br>New Zealand	An adaption of the Australian Centre for Education in Sleep (ACES) program - four 50-minute classroom-based education sessions with interactive groups along with a workbook with copies of the presentations and additional information.	Health education teacher at the participating school (trained by the program's developer)	**Bedtime:** No significant intervention effects for week nights, F(1, 24)=0.03, p=.88, and weekend nights, F(1,24)=0.16, p=.69<br>**Wake time:** No significant intervention effect for week nights, F(1, 24)=0.80, p=.38, and significant intervention effects (later wake times) for weekend nights favouring intervention condition, F(1, 24)=8.26, p=.01<br>**Total sleep time:** No significant intervention effect for week nights, F(1, 24)=0.96, p=.32, and intervention effect for weekend nights favouring intervention condition, F(1, 24)=5.21, p=.03<br>**Sleep hygiene:** No significant intervention effect, F(1, 24)=0.35, p=.56
Lin et al. (2018)[47]<br><br>Iran	Four 60min sessions with adolescents within the regular curriculum and one 60min session with their parents. The sessions were scheduled at approximately 2-week intervals and focused on behaviour change techniques targeting the theoretical determinants of sleep hygiene.	Two trained facilitators, each with a master's degrees in a relevant discipline and ≥10 years' experience of working with children and young people	**Sleep duration:** Significant intervention effects for weeknights favouring intervention condition (B=0.08; 95% CI: 0.001, 0.16), and no significant intervention effects for weekend nights (B=0.10; 95% CI: -0.37, 0.57)<br>**Sleep hygiene:** No significant intervention effects (B=0.07, 95% CI: -0.01, 0.15)
Lufi et al. (2011)[48]<br><br>Israel	Delayed school starting time of 1 hour.	Classroom teacher	**Sleep onset time:** No significant intervention effect (F=0.21, p≥.05, partial η^2=0.01)<br>**Sleep offset time:** Significant intervention effect (later sleep offset) favouring intervention condition (F=41.18, p<.01, partial η^2=0.71)<br>**Sleep duration:** Significant intervention effect favouring intervention condition (F=6.85, p<.05, partial η^2=0.29)<br>**Sleep efficiency:** No significant intervention effect (F=0.35, p=ns, partial η^2=0.02)
Moseley et al. (2009)[49]<br><br>Australia	Improving Adolescent Well-Being: Day and Night - four 50min classes using a cognitive-behaviour framework to promote well-being and healthy lifestyles (incorporating sleep-related components).	Researcher (a qualified teacher and registered psychologist)	**Sleep onset latency:** No significant intervention effects (all F ≤ 0.60, all p≥.44)<br>**Total sleep time:** No significant intervention effects (all F ≤ 0.60, all p≥.44)
Rigney et al. (2015)[50]<br><br>Australia	Australian Centre for Education in Sleep (ACES) program - four 50min classroom sessions at weekly intervals on sleep topics and an accompanying workbook with summaries of the lessons and educational activities. These sessions were followed by a group project on sleep topics, which was	Classroom teachers (trained by the program's developer)	***Actigraphy***<br>**Bedtime:** No significant intervention effect (p=.11)<br>**Sleep onset latency:** No significant intervention effect (p=.93)<br>**Wake time:** Significant intervention effect (p<.01) favouring

	presented at an evening education session that parents attended. Parents received a booklet about the ACES program with information and advice about how to implement the education into practice.		intervention condition by 10 minutes at 6 weeks, and no significant intervention effect at 18 weeks<br>**Total sleep time:** No significant intervention effect (p=.46)<br>**Sleep efficiency:** No significant intervention effect (p=.21)<br>***Self-report***<br>**Bedtime:** No significant intervention effect (p=.24)<br>**Wake time:** No significant intervention effect (p=.12)<br>**24 hour sleep:** Significant intervention effect (p<.02) favouring intervention condition by ~20 minutes at 6 weeks, and no significant intervention effect at 18 weeks<br>**Sleep hygiene:** No significant intervention effect (p=.19)
Sousa et al. (2013)[51]<br><br>Brazil	Five 45min meetings covering physiological and behavioural aspects of sleep and healthy lifestyles.	Researcher (a qualified middle and high school teacher)	**Bedtime:** Intervention effects unreported<br>**Wake time:** Intervention effects unreported<br>**Time in bed:** Intervention effects unreported
Tamura & Tanaka (2014)[52]<br><br>Japan	One 45min sleep education with self-help class (including 15min of self-assessment and 10min of instructions on completing a sleep diary), which focused on sleep hygiene and sleep improvement.	Sleep instructor	**Bedtime:** Significant intervention effects in favour of the intervention condition for children in 4th grade, $F(1,50)=6.70$, $p=.013$, 5th grade, $F(1,44)=8.03$, $p=0.007$, and 6th grade, $F(1,48)=5.37$, $p=0.025$<br>**Wake up time:** No significant intervention effects<br>**Sleep duration:** Significant intervention effect in favour of the intervention condition for children in 4th grade, $F(1,50)=4.99$, $p=0.03$, and no significant intervention effects for children in the 5th and 6th grades<br>**Sleep-related behaviours:** Intervention effects unreported
Tamura & Tanaka (2016)[53]<br><br>Japan	One 50min sleep education with self-help treatment class (including 25min of self-assessment), which focused on promoting regular sleep patterns and adjustment of biological rhythms.	Researcher (a registered psychologist and qualified sleep instructor)	**Bedtime:** Significant intervention effects in favour of the intervention condition for school nights, $F(1,241)=22.91$, $p<.001$, and weekend nights, $F(1,240)=12.12$, $p<.001$<br>**Sleep latency:** No results reported for school nights and a significant intervention effect in favour of the intervention condition for weekend nights, $F(1,240)=5.05$, $p=.026$<br>**Wake up time:** No significant intervention effects<br>**Total sleep time:** Significant intervention effects in favour of the intervention condition for school nights, $F(1,241)=14.45$, $p<.001$, and weekend nights, $F(1,241)=4.49$, $p=.035$<br>**Sleep promoting behaviours:** Significant intervention effects in favour of the intervention, $F(1,241)=15.71$, $p<.001$

Uhlig et al. (2019)[54]<br><br>The Netherlands	Rap & Sing Music Therapy - sixteen 45min weekly classes in which students developed individual and group rap themes and songs, and prepared them for audio and video recordings.	Qualified music therapists trained and supervised in Rap & Sing Music Therapy	**Sleep onset latency:** No significant intervention effect, F=0.14, p=.71, partial η^2=0.002<br>**Sleep time:** Significant intervention effects favouring the intervention condition, F=4.15, p<.05, partial η^2=0.05<br>**Sleep efficiency:** No significant intervention effect, F=0.03, p=.86, partial η^2<0.001<br>**Sleep episodes:** No significant intervention effect, F=0.10, p=.75, partial η^2=0.001<br>**Snooze time:** No significant intervention effect, F=1.88, p=.18, partial η^2=0.03
van Rijn et al. (2020)[55]<br><br>Singapore	Four 60min sleep education lessons on the importance of sleep, barriers to sufficient sleep, and time management to provide opportunities for sufficient sleep.	School principal and teachers, who received training on the intervention from the researchers	**Bedtime:** No significant intervention effect on school nights, F=2.62, p=.074, or weekend nights, F=0.07, p=.936<br>**Sleep onset latency:** No significant intervention effect on school nights, F=0.35, p=.703, or weekend nights, F=1.13, p=.325<br>**Wake up time:** No significant intervention effect on school nights, F=1.98, p=.140, or weekend nights, F=1.19, p=.307<br>**Total sleep time:** No significant intervention effect on school nights, F=2.33, p=.099, or weekend nights, F=1.13, p=.325<br>**Sleep efficiency:** No significant intervention effect on school nights, F=0.64, p=.053, and a significant intervention effect favouring intervention condition on weekend nights, F=4.26, p=.015
Wing et al. (2015)[57]<br><br>Hong Kong	Sleep education program including a town hall seminar, two 40min small class workshops (one per month), a slogan competition, a brochure, and an educational Web site. The program covered the importance of sleep, consequences of sleep deprivation, factors contributing to insufficient sleep, and good sleep practice. Sleep education seminars were offered to their parents and teachers.	Physicians experienced in sleep medicine and trained researchers	**Bedtime:** Significant intervention effect of -0:02 hours:minutes (95% CI: -0:03, 0:00) on weekdays favouring intervention condition and no significant intervention effect on weekends (difference in mean change=-0:01; 95% CI -0:06, 0:05)<br>**Wake time:** No significant intervention effect on weekdays (difference in mean change=0:01; 95% CI -0:00, 0:03) or weekends (difference in mean change=0:00; 95% CI -0:07, 0:07)<br>**Sleep duration:** Significant intervention effect of 0:03 hours:minutes (95% CI: 0:00, 0:06) on weekdays favouring intervention condition and no significant intervention effect on weekends (difference in mean change=0:01; 95% CI -0:05, 0:07)<br>**Caffeinated beverage consumption (≥3 times/week):** No significant intervention effects for adolescents who, at

			baseline, consumed beverages (OR=0.87; 95% CI: 0.61, 1.24) or did not consume beverages (OR=0.75; 95% CI: 0.50, 1.14)<br>**Alcohol consumption (≥3 times/week):** Intervention effects not calculatable for adolescents who, at baseline, consumed alcohol and no significant interaction effect for those who, at baseline did not consume alcohol (OR=0.37; 95% CI: 0.02, 6.23)<br>**Cigarette smoking (≥3 times/week):** No significant intervention effect for adolescents who, at baseline, smoked cigarettes (OR=1.38; 95% CI: 0.06, 33.18) or did not smoke cigarettes (OR=0.22; 95% CI: 0.02, 2.49)
Wolfson et al. (2015)[56]<br><br>United States	Sleep Smart Program - eight 40min sessions on adolescent sleep and strategies for healthy sleep hygiene practices and obtaining enough sleep. Booster sessions were held ~2 months and ~11 months after the sessions.	Pairs of trained facilitators, each with a Bachelor of Arts degree, who received supervision from a licensed clinical psychologist	**Bedtime:** Significant intervention effects favouring intervention condition for school nights (p≤.01) and weekend nights (p≤.01) at ~5 weeks and no significant intervention effects at ~10-11 months and ~15-16 months<br>**Wake time:** No significant intervention effects for school or weekend nights at ~5 weeks, ~10-11 months, and ~15-16 months<br>**Time in bed:** Significant intervention effects favouring intervention condition for school nights (p≤.05) and weekend nights (p≤.01) at ~5 weeks and no significant intervention effects at ~10-11 months and ~15-16 months<br>**Bedtime routine:** No significant intervention effects for school or weekend nights at ~5 weeks, ~10-11 months, and ~15-16 months<br>**Bedtime screen use:** Significant intervention effects favouring intervention condition at ~5 weeks (p≤.01) and no significant intervention effects at ~10-11 months and ~15-16 months<br>**PM caffeine use:** Significant intervention effects favouring intervention condition at ~5 weeks (p≤.05) and no significant intervention effects at ~10-11 months and ~15-16 months

Supplementary Table 2: Risk of bias.

Study	Domain 1/1a	Domain 1b	Domain 2	Domain 3	Domain 4	Domain 5	Overall
**Actigraphy Sleep Patterns**							
Beijamini & Louzada (2012)[58]	Some concerns	Some concerns	Low	Low	Low	Some concerns	Some concerns
Lufi et al. (2011)[48]	Some concerns	Some concerns	Low	High	Low	Some concerns	High
Rigney et al. (2015)[50]	Low	High	Low	Low	Low	Some concerns	High
Uhlig et al. (2019)[54]	Low	High	Low	Low	Low	Low	High
van Rijn et al. (2020)	Some concerns	Low	Low	Low	Low	Low	Some concerns
**Self-Reported Sleep Patterns**							
Baldursdottir et al. (2017)[38]	Some concerns	Low	Low	Low	Some concerns	Some concerns	Some concerns
Bavarian et al. (2016)[39]	Some concerns	Low	Low	Low	Some concerns	Some concerns	Some concerns
Bonnar et al. (2015)[40]	Some concerns	Some concerns	Low	Low	Some concerns	Some concerns	Some concerns
Cain et al. (2011)[41]	Some concerns	Low	Low	Low	Some concerns	Some concerns	Some concerns
Das-Friebel et al. (2019)[42]	Some concerns	Some concerns	Low	Low	Some concerns	Some concerns	Some concerns
Inhulsen et al. (2022)[43]	Some concerns	High	Low	Low	Some concerns	Some concerns	High
John et al. (2016)[44]	Some concerns	Some concerns	Low	Low	Some concerns	Some concerns	Some concerns
John et al. (2017)[45]	Some concerns	Some concerns	Low	Low	Some concerns	Some concerns	Some concerns
Kira et al. (2014)[46] and Blunden et al. (2012)[59]	Low	Not applicable	Low	Low	Some concerns	Some concerns	Some concerns
Lin et al. (2018)[47]	Low	Low	Low	Low	Some concerns	Some concerns	Some concerns
Moseley et al. (2009)[49]	Some concerns	Some concerns	Low	Low	Some concerns	Some concerns	Some concerns
Rigney et al. (2015)[50]	Low	High	Low	Low	Some concerns	Some concerns	High
Sousa et al. (2013)[51]	Some concerns	Not applicable	Low	Some concerns	Some concerns	Some concerns	Some concerns
Tamura & Tanaka (2014)[52]	Some concerns	Some concerns	Low	High	Some concerns	Some concerns	High
Tamura & Tanaka (2016)[53]	Low	Some concerns	Low	Low	Some concerns	Some concerns	Some concerns
Wing et al. (2015)[57]	High	Some concerns	Low	Some concerns	Some concerns	Some concerns	Some concerns
Wolfson et al. (2015)[56]	High	High	Low	Low	Some concerns	Some concerns	High
**Sleep Hygiene**							
Inhulsen et al. (2022)[43]	Some concerns	High	Low	Low	Some concerns	Some concerns	High

John et al. (2016)[44]	Some concerns	Some concerns	Low	Low	Some concerns	Some concerns	Some concerns
John et al. (2017)[45]	Some concerns	Some concerns	Low	Low	Some concerns	Some concerns	Some concerns
Kira et al. (2014)[46] and Blunden et al. (2012)[59]	Low	Not applicable	Low	Low	Some concerns	Some concerns	Some concerns
Lin et al. (2018)[47]	Low	Low	Low	Low	Some concerns	Some concerns	Some concerns
Rigney et al. (2015)[50]	Low	High	Low	Low	Some concerns	Some concerns	High
Tamura & Tanaka (2014)[52]	Some concerns	Some concerns	Low	High	Some concerns	Some concerns	High
Tamura & Tanaka (2016)[53]	Low	Some concerns	Low	Low	Some concerns	Some concerns	Some concerns
**Bedtime Routine**							
Wolfson et al. (2015)[56]	High	High	Low	Low	Low	Some concerns	High
**Bedtime Screen Use**							
Das-Friebel et al. (2019)[42]	Some concerns	Some concerns	Low	Low	Some concerns	Some concerns	Some concerns
Wolfson et al. (2015)[56]	High	High	Low	Low	Low	Some concerns	High
**Substance Use**							
Das-Friebel et al. (2019)[42]	Some concerns	Some concerns	Low	Low	Some concerns	Some concerns	Some concerns
Wing et al. (2015)[57]	Some concerns	Some concerns	Low	Some concerns	Some concerns	Some concerns	Some concerns
Wolfson et al. (2015)[56]	High	High	Low	Low	Low	Some concerns	High

Note. Domain 1/1a: Risk of bias arising from the randomisation process. Domain 1b: Risk of bias arising from the timing of identification or recruitment of participants in a cluster-randomized trial. Domain 2: Risk of bias due to deviations from the intended interventions. Domain 3: Risk of bias due to missing outcome data. Domain 4: Risk of bias in measurement of the outcome. Domain 5: Risk of bias in selection of the reported result. Overall: Overall risk of bias.
